# SUrvey of renal Biopsy registry database and Anticancer dRUg therapy in Japan (SUBARU-J study)

**DOI:** 10.1093/ckj/sfae327

**Published:** 2024-11-28

**Authors:** Takashige Kuwabara, Yoshikazu Miyasato, Tomoko Kanki, Teruhiko Mizumoto, Takeshi Matsubara, Naoki Sawa, Hitoshi Sugiyama, Shoichi Maruyama, Hiroshi Sato, Tatsuo Tsukamoto, Tomohiro Murata, Mariko Miyazaki, Toshiyuki Imasawa, Masashi Mukoyama, Naoka Murakami, Kenar D Jhaveri, Motoko Yanagita, the JSN Onconephrology working group, Motoko Yanagita, Haruna Kawano, Takehiko Kawaguchi, Takashige Kuwabara, Kenichiro Koitabashi, Naoki Sawa, Takeshi Matsubara, Shinichi Mizuno, Takehiko Wada

**Affiliations:** Department of Nephrology, Kumamoto University Graduate School of Medical Sciences, Kumamoto, Japan; Department of Nephrology, Kumamoto University Graduate School of Medical Sciences, Kumamoto, Japan; Department of Nephrology, Kumamoto University Graduate School of Medical Sciences, Kumamoto, Japan; Department of Nephrology, Kumamoto University Graduate School of Medical Sciences, Kumamoto, Japan; Department of Nephrology, Kyoto University Graduate School of Medicine, Kyoto, Japan; Nephrology Center, Toranomon Hospital Kajigaya, Kanagawa, Japan; Department of Medicine, Kawasaki Medical School General Medical Center and Department of Medical Care Work, Kawasaki College of Allied Health Professions, Okayama, Japan; Department of Nephrology, Nagoya University Graduate School of Medicine, Nagoya, Japan; Department of Nephrology, Rheumatology and Endocrinology, Tohoku University Graduate School of Medicine, Sendai, Miyagi, Japan; Department of Nephrology and Dialysis, Medical Research Institute Kitano Hospital, PIIF Tazuke-Kofukai, Osaka, Japan; Department of Cardiology and Nephrology, Mie University Graduate School of Medicine, Mie, Japan; Department of Nephrology, Rheumatology and Endocrinology, Tohoku University Graduate School of Medicine, Sendai, Miyagi, Japan; Department of Nephrology, National Hospital Organization Chibahigashi National Hospital, Chiba, Japan; Department of Nephrology, Kumamoto University Graduate School of Medical Sciences, Kumamoto, Japan; Division of Nephrology, Washington University in St. Louis, St. Louis, MO, USA; Glomerular Center at Northwell Health, Division of Kidney Diseases and Hypertension, Northwell Health, Great Neck, NY, USA; Department of Nephrology, Kyoto University Graduate School of Medicine, Kyoto, Japan

**Keywords:** anticancer drug therapy, kidney biopsy registry database, PPI use, thrombotic microangiopathy

## Abstract

**Background:**

Kidney complications associated with anticancer drug therapy have greatly increased recently. We aimed to investigate the real-world clinical outcomes of anticancer drug therapy–associated renal complications in Japan using the national kidney biopsy database, Japan Renal Biopsy Registry (J-RBR).

**Methods:**

From 2018 to 2021, 449 cases from 49 facilities identified as ‘drug-induced’ histopathology in the J-RBR were screened, of which a total of 135 were confirmed as anticancer drug–related cases and included in the analysis. Overall survival rates were estimated using the Kaplan–Meier method and compared by logrank test. The Cox regression model was used to evaluate the association between variables and deaths.

**Results:**

The most common primary sites of malignancies were the lung (33.3%), followed by gastrointestinal (16.3%) and gynaecological (11.1%) cancers. Tubulointerstitial nephritis (TIN; 47.4%) and thrombotic microangiopathy (TMA; 35.6%) were the most frequent diagnoses. All immunoglobulin A nephropathy, minimal change disease and crescentic glomerulonephritis (CrGN) cases were immune checkpoint inhibitor related. All CrGN cases were anti-neutrophil cytoplasmic antibody negative. Antibiotics were most frequently used concomitantly with anticancer drugs in TMA cases among subgroups (TMA versus others: 62.5 versus 27.5%; *P* < .001). Among TMA cases, the serum lactate dehydrogenase level tended to be higher in cytotoxic agent–associated TMA (CTx-TMA) than in other TMAs, but was not significant between groups (415.5 versus 219.0 U/l; *P* = .06). Overall survival was worse in CTx-TMA than in other TMAs (*P* = .007). The Cox model demonstrated proton pump inhibitor (PPI) use (hazard ratio 2.49, *P* = .001) as a significant prognostic factor, as well as the presence of metastasis and serum albumin level.

**Conclusions:**

Our registry analysis highlighted various presentations of biopsy-proven kidney complications associated with anticancer drug therapy. Clinicians should be aware of worse outcomes associated with CTx-TMA and the prognostic role of PPI use.

## Graphical Abstract

**Graphical Abstract:**
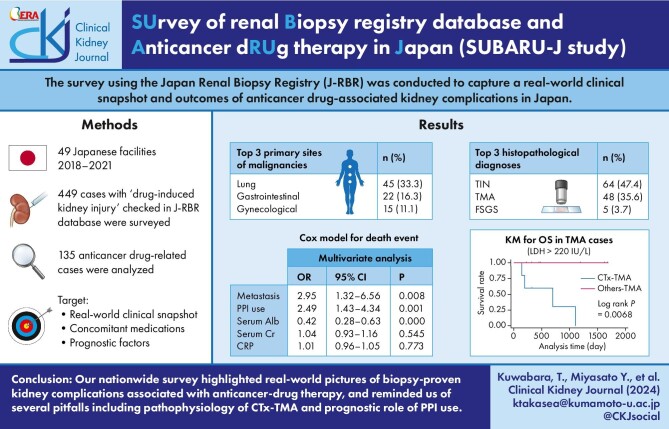

KEY LEARNING POINTS **What was known:**

- Kidney injury is one of the critical adverse effects associated with anticancer therapy and can affect the overall prognosis and management of patients with cancer.
- Among biopsy-proven pathology, thrombotic microangiopathy (TMA) has been well studied as an anticancer therapy–associated complication, but clinical variables associated with kidney outcomes were unclear.
- Proton pump inhibitor (PPI) use was reported as a significant risk factor associated with immune-related adverse event tubulointerstitial nephritis (TIN) in several previous reports, but its roles in other anticancer-related therapies are unknown.

**This study adds:**

- TIN and TMA were the most frequent diagnoses, and all immunoglobulin A nephropathy, minimal change disease and crescentic glomerulonephritis cases were immune checkpoint inhibitor related.
- There is a significant difference in the prognosis between cytotoxic agent–associated TMA (CTx-TMA) and elevated lactate dehydrogenase TMA cases.
- PPI use is a significant prognostic factor as well as presence of metastasis and serum albumin level.

**Potential impact:**

- This is the first nationwide registry of anticancer drug–related kidney injury using the Japan Renal Biopsy Registry, one of the largest national registries of kidney biopsies.
- This study reminded us of several pitfalls, including the pathophysiology of CTx-TMA and the prognostic role of PPI use.

## INTRODUCTION

Cancer treatment has advanced tremendously over the past few decades, with the development of various anticancer drugs that target specific mechanisms involved in tumour growth/proliferation and the immune system. While these therapies have improved survival rates and quality of life for many patients with cancer, they are not without side effects. One of the critical adverse effects associated with these treatments is kidney injury, which can compromise renal function and affect the overall prognosis and management of patients with cancers [1, 2]. Anticancer drug therapy–associated kidney injury encompasses a range of renal complications that arise due to the nephrotoxic effects of chemotherapy agents, targeted therapies, immunotherapies and other related drugs. These injuries can manifest in several forms, including acute kidney injury (AKI), chronic kidney disease (CKD), electrolyte imbalances, acid–base disorders and hypertension. The pathophysiology behind these renal complications is complex and involves direct tubular toxicity, altered renal haemodynamics and immune-mediated damage. Kidney biopsy is the gold standard to make a histological diagnosis, while in recent years, empiric treatment of certain conditions, such as in typical tubulointerstitial nephritis (TIN) of immune-related adverse events (irAEs), is often recommended. However, especially those involving glomerular or microvascular lesions, it would be meaningful to consider kidney biopsy for pathological details. In particular, thrombotic microangiopathy (TMA), including kidney-limited TMA, is a potentially serious complication related to anticancer therapy that can only be diagnosed by biopsy, and the pathophysiology may differ depending on the causative agent [3–5]. Although several case reports and systematic reviews can be found, there are few comprehensive reports based on kidney biopsy diagnoses.

The Japanese Society of Nephrology (JSN) established a Japanese nationwide registry of kidney biopsies [Japan Renal Biopsy Registry (J-RBR)] in 2007 [6], and this registry was revised and expanded in 2018 [7]. This updated J-RBR has become one of the largest nationwide registries for kidney diseases, notably including additional information on the causative agents for drug-induced kidney diseases [8]. In this study, we conducted a cohort study using the updated J-RBR to understand the real-world clinical practice and outcomes of anticancer drug therapy–associated kidney complications in Japan.

## MATERIALS AND METHODS

### Selection of the patients from J-RBR

**Figure 1:**
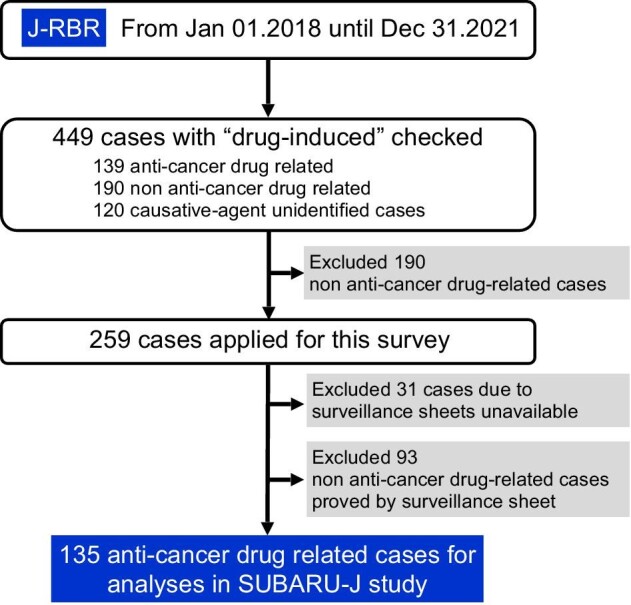
Flow chart of patient selection for final analysis.

**Figure 2:**
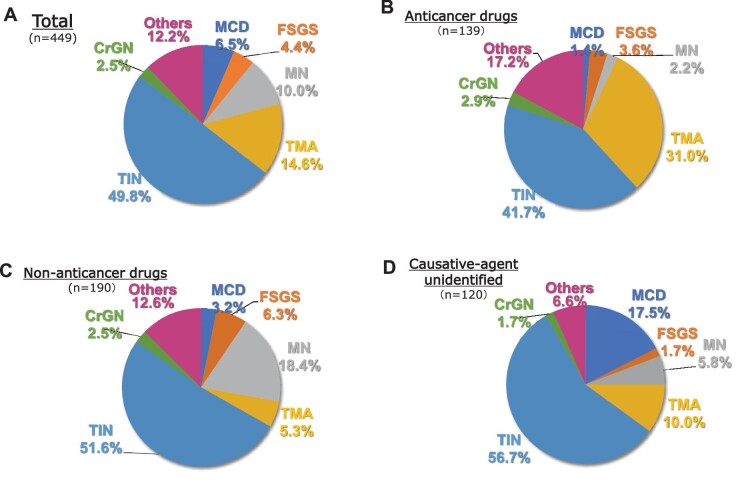
Distribution of the histopathological diagnoses by a causative agent. Pie charts show the histopathological distributions in **(A)** total subjects and subgroups of **(B)** anticancer drugs, **(C)** non-anticancer drugs and **(D)** causative agent unidentified. FSGS: focal segmental glomerulosclerosis.

The patient selection strategy is shown in Fig. 1. Detailed clinical information was collected from 449 ‘drug-induced’ cases reported in the J-RBR database from 2018 to 2021 in 47 nephrology centres (Appendix S2). TIN was the most common histopathological diagnosis, regardless of the causative agent (Fig. 2A–D). However, the second most common diagnosis was TMA in the anticancer drug subgroup and membranous nephropathy (MN) in the non-anticancer drug subgroup (Fig. 2B and C). After excluding 190 cases that were ‘non-anticancer drug-related’, additional clinical data were further collected from corresponding institutions in 259 cases (Fig. 1). Data included renal pathology, medications and cancer prognosis, if they had a history of cancer. A total of 135 anticancer drug–related cases confirmed by the information were analysed. Patients’ clinical data collected pre- and post-biopsy, as well as histopathological diagnoses, were used for epidemiological and clinicopathological analyses.

### Clinical data

Patient demographics and the following clinical parameters were collected from the J-RBR database registered by the attending nephrologist at the time of the kidney biopsy: body mass index (BMI), diabetes, antihypertensive drug use, systolic and diastolic blood pressure (SBP/DBP), causative agents, serum total protein, serum albumin, haemoglobin A1c (HbA1c), serum creatinine, estimated glomerular filtration rate (eGFR; calculated using age, sex and serum creatinine at biopsy according to the estimation equation for Japanese adults [9]), C-reactive protein (CRP), urine protein:creatinine ratio in all subjects and anti-neutrophil cytoplasmic antibody (ANCA) titres in subjects with crescentic glomerulonephritis (CrGN). In addition to these data, the following additional information was obtained: detailed anticancer causative agents, concomitant medications [proton pump inhibitor (PPI), non-steroidal anti-inflammatory drugs (NSAIDs), renin–angiotensin system inhibitor (RASi), H_2_ blocker, allopurinol, antibiotics], primary site of malignancies, cancer prognosis according to the RECIST 1.1 criteria [10], overall survival referring to the length of time from the kidney biopsy in all subjects and platelet counts and serum lactate dehydrogenase (LDH) in subjects with TMA.

### Histopathological data

Data on the histopathological diagnoses were obtained from the J-RBR database registered by the nephropathologists.

### Statistical analyses

Data are expressed using mean [standard deviation (SD)] or median [interquartile range (IQR)] for continuous variables and percentages for discrete variables, as appropriate. The Mann–Whitney U test was used for comparison of continuous variables. The chi-squared test or Fisher's exact test was used for comparison of frequencies. Overall survival rates between anticancer drug groups were estimated using the Kaplan–Meier method and the survival estimates were compared by logrank test. *P*-values <.05 were regarded as statistically significant. For evaluating the association between variables and deaths, we used Cox regression models. The proportional hazards assumption was checked by Schoenfeld residuals. All analyses were carried out with Stata BE version 18 (StataCorp, College Station, TX, USA).

## RESULTS

### Clinical characteristics of anticancer drug–associated cases

The clinical characteristics of those enrolled in the SUrvey of renal Biopsy registry database and Anticancer dRUg therapy in Japan (SUBARU-J) study are listed in Table 1. Males are dominant (60.7%) and 26% and 59% of the subjects had diabetes and were on antihypertensive therapy, respectively. The most common primary site of malignancies was the lung, followed by gastrointestinal and gynaecological cancers. The two most common causative agents were programmed cell death 1 (PD-1) inhibitor and vascular endothelial growth factor monoclonal antibody (VEGF mAb), the main causes of TIN and TMA, respectively. The frequency of concomitant medications was 45.2% for PPIs, 14.8% for NSAIDs and 40.0% for antibiotics. The associations of this concomitant medication use and histopathological diagnoses will be described later (Table 2). The renal function determined by eGFR was 27.4 ml/min/1.73 m^2^ (median).

**Table 1:** Subject characteristics (*N* = 135).

Characteristics	Values
Age (years), median (IQR)	68 (60–74)
Sex, *n* (%)	
Male	82 (60.7)
Female	53 (39.3)
BMI (kg/m^2^), median (IQR)	22.1 (19.7–24.7)
Diabetes mellitus, *n* (%)	34 (26.0)
Antihypertensive drug use, *n* (%)	80 (59.3)
Systolic blood pressure (mmHg), mean (SD)	131.7 (17.4)
Diastolic blood pressure (mmHg), mean (SD)	75.1 (12.6)
Primary site of malignancy, *n* (%)	
Lung	45 (33.3)
Gastrointestinal	22 (16.3)
Gynaecological	15 (11.1)
Hepatobiliary pancreas	12 (8.9)
Head and neck	10 (7.4)
Urological	9 (6.7)
Blood	5 (3.7)
Breast	5 (3.7)
Brain	2 (1.5)
Bone and soft tissue	1 (0.7)
Other	9 (6.7)
Causative agents, *n* (%)	
PD-1 inhibitor	46 (34.1)
VEGF mAb	24 (17.8)
ICI combination	10 (7.4)
Multikinase TKI	9 (6.7)
VEGFR2 mAb	9 (6.7)
Antimetabolites	8 (5.9)
Platinum-based	8 (5.9)
BCR-ABL TKI	5 (3.7)
PD-L1 inhibitor	5 (3.7)
PD-L1i + VEGF mAb combination	4 (3.0)
EGFR TKI	3 (2.2)
Alkylating agents	1 (0.7)
Aromatase inhibitor	1 (0.7)
Other	2 (1.5)
Cancer prognosis, *n* (%)	
Complete response	21 (15.6)
Partial response	21 (15.6)
Stable disease	21 (15.6)
Progressive disease	63 (46.7)
Unknown	9 (6.7)
Metastasis of cancer, *n* (%)	100 (74.1)
Concomitant medication, *n* (%)	
RAAS inhibitor use	5 (3.7)
PPI use	61 (45.2)
H2 blocker use	5 (3.7)
NSAIDs use	20 (14.8)
Allopurinol use	5 (3.7)
Antibiotics use	54 (40.0)
Laboratory variables at renal biopsy	
Blood test	
Total protein (g/dl), median (IQR)	6.5 (5.9–7.2)
Albumin (g/dl), mean (SD)	3.3 (0.7)
Creatinine (mg/dl), median (IQR)	1.7 (1.1–3.2)
eGFR (ml/min/1.73 m^2^), median (IQR)	27.4 (15.3–48.1)
CRP (mg/dl), median (IQR)	0.4 (0.1–2.2)
HbA1c (%),median (IQR)	5.8 (5.4–6.3)
Urinary test	
Urine protein:creatinine (g/gCr), median (IQR)	1.3 (0.3–4.4)

BMI: body mass index.

**Table 2:** Histopathological types and concomitant medications.

Medications	TIN (*n* = 64)	TMA (*n* = 48)	Other (*n* = 23)	*P*-value
PPI, *n* (%)	33 (51.6)	16 (33.3)	12 (52.2)	.128
H2 blocker, *n* (%)	3 (4.7)	1 (2.1)	1 (4.4)	.714
RAASi, *n* (%)	2 (3.1)	2 (4.2)	1 (4.4)	1.000
NSAIDs, *n* (%)	13 (20.3)	5 (10.4)	2 (8.7)	.304
Allopurinol, *n* (%)	2 (3.1)	2 (4.2)	1 (4.4)	1.000
Antibiotics, *n* (%)	15 (23.4)	30 (62.5)	9 (39.1)	.000

RAASi: renin–angiotensin–aldosterone system inhibitor.

### Histopathological types and their causative agents

Histopathologically, TIN and TMA were the most frequent diagnoses (Table 3). Of TIN, 80% were immune checkpoint inhibitor (ICI) related and 75% of TMA were associated with anti-angiogenic therapy. Of note, all immunoglobulin A nephropathy (IgAN), minimal change disease (MCD) and CrGN cases were ICI related. There was no ANCA-positive case in CrGN (data not shown). Supplementary Table S1 describes more detailed information on anticancer drugs.

**Table 3:** Histopathological types and their causative agents (N = 135).

Types and agents	Values
TIN, *n* (%)	64 (47.4)
CTx	9 (14.1)
ICI	21 (32.9)
CTx + ICI	28 (43.8)
CTx + AAG	3 (4.7)
ICI + AAG	2 (3.1)
Other	1 (1.6)
TMA, *n* (%)	48 (35.6)
CTx	6 (12.5)
ICI	1 (2.1)
Anti-AG	9 (18.8)
CTx + AAG	24 (50.0)
ICI + AAG	2 (4.2)
Other	6 (12.5)
FSGS, *n* (%)	5 (3.70)
CTx	1 (20.0)
AAG	3 (60.0)
Other	1 (20.0)
IgAN, *n* (%)	4 (2.96)
ICI	3 (75.0)
CTx + ICI	1 (25.0)
ATN, *n* (%)	3 (2.22)
CTx	2 (66.7)
CTx + ICI	1 (33.3)
MCD, *n* (%)	3 (2.22)
ICI	3 (100.0)
CrGN, *n* (%)	2 (1.48)
ICI	1 (50.0)
ICI + AAG	1 (50.0)
MPGN, *n* (%)	2 (1.48)
ICI	1 (50.0)
CTx + AAG	1 (50.0)
AMy, *n* (%)	1 (0.74)
Other	1 (100.0)
C3GN, *n* (%)	1 (0.74)
Other	1 (100.0)
DKD, *n* (%)	1 (0.74)
ICI + AAG	1 (100.0)
NScl, *n* (%)	1 (0.74)
CTx + Other	1 (100.0)

FSGS: focal segmental glomerulosclerosis; MPGN: membranoproliferative glomerulonephritis; Amy: amyloidosis; C3GN: C3 glomerulonephritis; DKD: diabetic kidney disease; NScl: nephrosclerosis; AAG: anti-angiogenic agent.

### Associations between concomitant medication and histopathological categories

Since PPI use was reported as a significant risk factor associated with irAE-TIN [11–14], we aimed to determine whether concomitant medications may be associated with drug-induced kidney injuries. The concomitant medications by histopathological diagnoses are summarized in Table 2. In our study, there was no high-frequency use of PPI or NSAIDs, another risk for allergic TIN, in the TIN group compared with the other histopathological categories. On the other hand, antibiotic use was more prevalent in the TMA group (TMA versus others: 62.5 versus 27.5%; *P* < .001). Supplementary Table S2 describes more detailed information on histopathological types.

### Overall survival in TIN and TMA among causative agent subgroups

**Figure 3:**
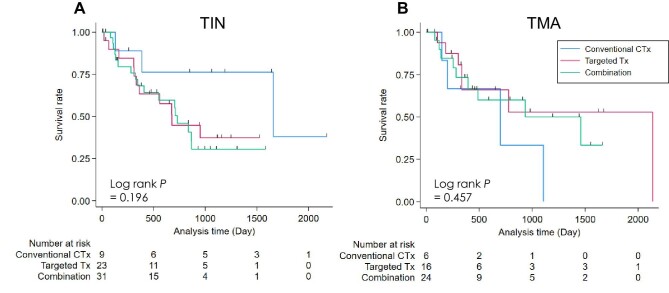
Kaplan–Meier curves for overall survival in (A) TIN and (B) TMA. Cases with missing overall survival data were excluded from this analysis. Conventional CTx: conventional cytotoxic agents; targeted Tx: targeted therapy.

Among cases with TIN, their overall survival (OS) in the subgroups of targeted therapy (targeted Tx), and the combination with targeted and conventional cytotoxic agents (combination) seemed to be worse than that of conventional cytotoxic agents alone (conventional CTx) (Fig. 3). It may be because cancer progression was more frequent in TIN cases who received targeted Tx and combination (Supplementary Table S3). In contrast with TIN, among TMA cases, OS in each of the three subgroups was similar, suggesting the prognostic impact of conventional CTx-TMA on OS.

### Serum LDH levels and overall survival in TMA between CTx-TMA and others-TMA

**Figure 4:**
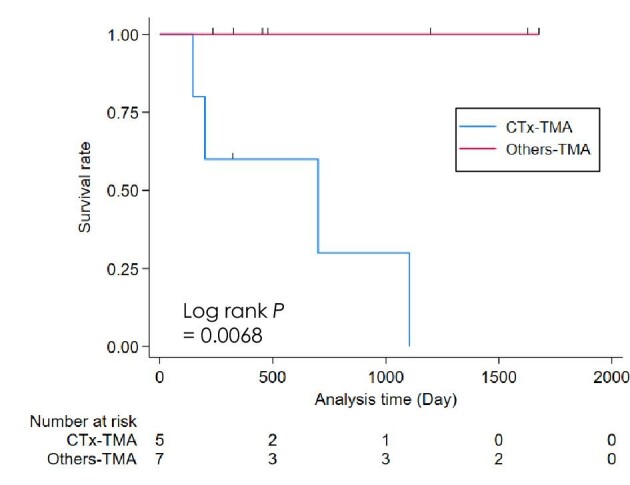
Kaplan–Meier curves for overall survival in the CTx-TMA and others-TMA subgroups in elevated LDH TMA cases. TMA cases with LDH >220 IU/l were employed in this analysis. Cases in a subgroup with a combination treatment of cytotoxic agents and anti-angiogenic agents (CTx + AAG) were excluded from this analysis.

The serum LDH level tended to be higher in CTx-TMA than in others-TMA, but not significant between groups (Table 4). Others-TMA is mainly due to anti-angiogenic therapies (Supplementary Table S4). This result is consistent with a recent registry cohort [5]. Of note, even in the TMA cases with high LDH (>220 IU/l), there is a significant difference in the prognosis between CTx-TMA and the others-TMA (Fig. 4). There appears to be no apparent substantial gap in cancer prognosis between these two groups (Supplementary Table S5).

**Table 4:** Serum LDH and platelet count in the CTx-TMA and others-TMA groups.

	Overall ( *n* = 23)	CTx-TMA (*n* = 6)	Others-TMA (*n* = 17)	*P*-value
Serum LDH (IU/l), median (IQR)	222 (183 362)	415.5 (224 519)	219 (183 237)	.059
Platelet count (10^3^/μl), median (IQR)	174 (116 215)	109.5 (74 192)	178 (146 215)	.286

## DISCUSSION

In this nationwide cohort study of the J-RBR, we demonstrated the distribution of biopsy-proven histopathology and concomitant medications in anticancer drug–associated kidney complications. Furthermore, it is important to note the potential prognostic impact of differences in causative agents and concomitant medications. The renal biopsy registry (J-RBR) was established in 2007 [6] and evolved to include information on the causative drug in 2018 [7, 8]. The frequency of renal complications associated with anticancer drug therapy has greatly increased in recent years and this is evident from the yearly registration numbers in the J-RBR: 202 anticancer therapy–associated cases were registered between 2018 and 2020, while 247 cases were added in 2021 alone, achieving a total number of 449 cases in this study. To date, there have been no previous reports on anticancer drug–associated kidney events using a nationwide renal biopsy registry, partly because there were insufficient numbers of cases in each institution. Our nationwide survey using the updated J-RBR provides a real snapshot of kidney involvement associated with anticancer drug therapy in Japan.

The primary site of malignancies is similar to the global trend in the number of patients with cancer [15, 16], which might reflect the cancer populations in each organ. Lung, colorectal and breast cancers are increasing worldwide. This might have increased the number of early diagnoses for these cancers, probably due to their becoming curable through advances in molecular targeted therapies. However, there is a specific feature of the histological diagnoses, in which TIN and TMA are the most prevalent. The treatment regimen employed in these top three cancers may also have an impact. We should consider selection bias in that a kidney biopsy is more likely to be performed in the cases suspected of TIN, TMA and also nephrotic syndrome. Also, since the first-line treatment regimen for lung cancer, the top-ranked in our survey, usually includes platinum-based chemotherapy, we have to consider the side effects of the first-line regimen on the renal prognostic role as well as chronic tubulointerstitial changes.

Despite the small number of cases, it should be noted that all IgAN, MCD and CrGN cases were associated with ICI in our study. A previous systematic review also reported a variety of types of glomerular diseases induced by ICI [17]. As reported in other reports, all CrGN cases in our cohort were ANCA negative. It is also important that all MCD cases are ICI related. We usually assume that the nephrotic syndrome cases treated with the combination therapy of ICI and anti-angiogenic therapy might be caused by anti-angiogenic agents targeting VEGF signalling. However, our results caution that the possibility of nephrotic syndrome caused by ICI in such cases should be kept in mind. There are several reports suggesting that AKI development is a mortality risk factor in patients on cancer treatment [18, 19]. Although serum creatinine at the time of kidney biopsy was a significant factor in our study in univariate analysis (Supplementary Table S6), it did not remain a significant risk factor [11] after adjustment for confounding factors. It might be due to the small sample number or varied causative agents. Meanwhile, the known poor prognostic factor, serum albumin, was identified as an independent and significant factor in the multivariate analysis, suggesting the validity of our database (Supplementary Table S6).

Although a higher frequency of PPI use in TIN was not observed in our total subjects rather than in ICI-related cases, it has been established as a significant risk factor associated with irAE-TIN [11–14]. We will focus on ICI-treated cases for the next issue. Recently, it has been reported that a history of PPI use in ICI-treated patients is associated not only with renal complications, but also with worsening progression-free survival and OS [20], prompting cautious attention. Notably, PPI use remained a significant factor in our Cox multivariate analysis as well as cancer metastasis and serum albumin level (Supplementary Table S6). PPI use might provide negative aspects partly through affecting the gut microbiota [21, 22]. Furthermore, it is a clinically suggestive finding that antibiotic use was clearly higher in the TMA group, and we should mention several possibilities in a TMA case treated with anticancer agents and antibiotics as follows. Neutrophil extracellular traps (NETs) released from activated neutrophils may contribute to endothelial dysfunction and thrombosis formation during infection [23, 24]. Some antibiotics potentially cause TMA [4, 25]. Additionally, complement-mediated TMA, also previously called atypical haemolytic uraemic syndrome, might be triggered by infection or antibiotics [4, 25].

A recent French registry cohort study reported that a higher incidence of systemic TMA and renal-limited TMA are associated with CTx-TMA and antiangiogenic-associated TMA (AAG-TMA), respectively [5]. Furthermore, there is no platelet thrombi but hyaline pseudo-thrombi observed in AAG-TMA, which should be categorized as glomerular microangiopathy (GMA) rather than TMA [26, 27]. In our study, the result of serum LDH also supports these reports. It is noteworthy that the distinct prognosis between CTx-TMA and others-TMA suggests the pathophysiology itself might be different. Although CTx-TMA, including gemcitabine associated, usually arises due to dose-dependent toxicity, single-dose or low-dose gemcitabine might cause TMA, particularly with other predisposing factors [28, 29]. Hence, gemcitabine-induced TMA probably involves immune- and non-immune-mediated mechanisms [30]. There was no case treated with eculizumab, a monoclonal antibody against C5 complement factor, in CTx-TMA in our study. However, eculizumab might be potentially effective on gemcitabine-induced TMA [31, 32]. Our results indicated a poor prognosis in CTx-TMA compared with others-TMA, even if in the LDH-elevated cases for both TMA groups. The potential anti-complement therapy for this entity should be investigated [33].

There are several limitations to be noted in our study. We cannot exclude the selection bias that kidney biopsy may be performed in cases with an expected favourable prognosis. It should be noted that the survival time analysis might not represent the original population, due to the small number of cases. Also, mortality events reflect all-cause death, not only cancer death.

Our SUBARU-J study has several advantages and strengths even with some of the above limitations. This is the first nationwide survey of anticancer drug–related renal injury using the J-RBR, one of the largest national registries of kidney biopsies. Renal complications in onconephrology are quite fruitful, although the number of cases experienced by each nephrologist is limited in each single facility. Therefore, a strategy of this survey including detailed information about drug-induced kidney diseases was the most suitable way to achieve our aim, highlighting real-world cases in our clinical practice and also reminding us of several pitfalls. Furthermore, a small retrospective cohort of TMA cases prompted us to consider the pathophysiological details among the causative anticancer agents even in the same category of conventional histological diagnosis. To explore the details of anticancer drug–associated TMA and other complications, further research employing a larger sample size and also basic research are warranted.

## Supplementary Material

sfae327_Supplemental_Files

## Data Availability

The data underlying this article are available in the text and its online supplementary material. Restrictions apply to the availability of these data, which were used under license for this study. The original full dataset analysed for this study is available with the permission of the JSN. The interpretation and reporting of these data are the responsibility of the authors and in no way should be seen as an official policy or interpretation of the JSN.

## Notes

Members of the JSN Onconephrology working group are listed in Appendix S1. These members are collaborators unless also mentioned in the author list above, in which case they are authors.
